# Supplementary material for: Insights into the Datasets, Tools, and Training Needs of the AnVIL Community: 2024
Source: bioRxiv. 2025 Nov 17:2025.11.14.688517. Preprint. [Version 1] doi: 10.1101/2025.11.14.688517 (PMC12667874; doi:10.1101/2025.11.14.688517)
Supplement: Supplement 1 [file media-1.pdf]

		Examine the background and current work of users to develop appropriate personas	Understand barriers to platform adoption and user preferences for training and support	Assess researchers' technological comfort with cloud-based genomic analysis tools	Identify computational and data analysis resource needs
Part 1: Self-identify		*x*			
	How would you describe your current usage of the AnVIL platform?	*x*			
Part 2: Feature importance + Returning user specific Q’s		*x*	*x*	*x*	*x*
	Rank features according to importance		*x*		*x*
	Knowledge of tech/data resources on the AnVIL			*x*	
	Length of use	*x*			
	Favorite AnVIL feature	*x*			
	Foreseeable computational/storage needs				*x*
	Recommendation likelihood	*x*			
Part 3: Demographics		*x*			
	Highest Degree	*x*			
	Industry	*x*			
	Kind of work	*x*			
	Institutional affiliation	*x*			
	Consortia affiliations	*x*			
Part 4: Experience		*x*		*x*	*x*
	Tech/data resource knowledge separate from the AnVIL			*x*	
	Types of data analyzed				*x*
	Experience with human clinical, human genomic, or non-human genomic data	*x*			
	General & specific interest in controlled access datasets				*x*
Part 5: Awareness			*x*		
	Monthly AnVIL Demos		*x*		
	AnVIL Support		*x*		
Part 6: Preferences			*x*	*x*	*x*
	Rank various training workshop modalities		*x*		
	Where analyses are currently run			*x*	*x*
	DMS compliance/data repositories				*x*
	Source of funds for cloud computing		*x*		

Supplemental Table 1

Relation of study aims to the design of the State of the AnVIL 2024 Community Poll (broken down by section and question).

Sections of the State of the AnVIL 2024 Community Poll (Part 1, Part 2, etc.) are listed in the first column as the row names. Column 2 provides the questions for each part. The rest of the column names are the enumerated study aims. X’s are added at the intersection of any questions relevant to a particular study aim.
