# Supplementary material for: Insights into the Datasets, Tools, and Training Needs of the AnVIL Community: 2024
Source: bioRxiv. 2025 Nov 17:2025.11.14.688517. Preprint. [Version 1] doi: 10.1101/2025.11.14.688517 (PMC12667874; doi:10.1101/2025.11.14.688517)
Supplement: Supplement 2 [file media-2.pdf]

Responses	Support Option	Awareness	Use (Past or Future)	Use (Past only) Demo: "Attend"
No, didn't know of	Demo			
	Support Forum			
No, but aware of	Demo	x		
	Support Forum	x		
Not yet, but am registered to	Demo	x	x	
Yes, one	Demo	x	x	x
Yes, multiple	Demo	x	x	x
Answered someone's post	Support Forum	x	x	x
Posted in	Support Forum	x	x	x
Read through other's posts	Support Forum	x	x	x

Supplemental Table 2

Recategorization of AnVIL Demo and Support Forum Utilization Raw Responses into Awareness and Use

This table provides all possible responses to the two questions asking respondents about their utilization of the AnVIL Demos and Support Forum. The second column describes which responses are applicable for each support option. The last 3 columns use an x if the raw response aligns with awareness or use.
